# Effects of Resveratrol on Daily Rhythms of Locomotor Activity and Body Temperature in Young and Aged Grey Mouse Lemurs

**DOI:** 10.1155/2013/187301

**Published:** 2013-07-25

**Authors:** Fabien Pifferi, Alexandre Dal-Pan, Solène Languille, Fabienne Aujard

**Affiliations:** Mécanismes Adaptatifs et Evolution, UMR 7179 Centre National de la Recherche Scientifique, Muséum National d'Histoire Naturelle, 1 avenue du Petit Château, 91800 Brunoy, France

## Abstract

In several species, resveratrol, a polyphenolic compound, activates sirtuin proteins implicated in the regulation of energy balance and biological clock processes. To demonstrate the effect of resveratrol on clock function in an aged primate, young and aged mouse lemurs *(Microcebus murinus)* were studied over a 4-week dietary supplementation with resveratrol. Spontaneous locomotor activity and daily variations in body temperature were continuously recorded. Reduction in locomotor activity onset and changes in body temperature rhythm in resveratrol-supplemented aged animals suggest an improved synchronisation on the light-dark cycle. Resveratrol could be a good candidate to restore the circadian rhythms in the elderly.

## 1. Introduction

Aging is associated with changes in circadian rhythmicity of endocrine, metabolic, and behavioral properties in several mammalian species [1]. It has important effect on thermoregulation processes [2] and on locomotor activity [3, 4]. 

Grey mouse lemur (*Microcebus murinus*, a Malagasy nonhuman primate) is a nocturnal species exhibiting high levels of locomotor activity during the dark period and a complete rest during the light period. Under exposure to short days, daily rhythm of body temperature (Tb) in mouse lemur is characterized by high values during the active period and high levels of locomotor activity (LA) and, before the onset of the light phase, a rapid and linear drop in Tb and LA, reaching minimal value after 3-4 hours. This hypothermia bout is followed by a spontaneous rewarming to normothermic Tb levels until the following dark phase. Tb, LA, and hypothermia are thus closely related and all driven by circadian regulation. In this species, age-related physiological changes have been identified (see [5] for review). Among these changes, an age-related decrease in amplitude of the seasonal rhythms of body mass, basal metabolic rate, and testosterone has been demonstrated [6]. More particularly, the daily rhythm of LA is fragmented and the amplitude of movement is reduced in aged animals compared to young [7, 8]. Thermoregulation processes are also impaired during aging in this species. During winter season, when ambient temperature is low, aged animals exhibit deeper hypothermia and increased levels of energy expenditure, impairing energy balance [9]. Aging is also known to shorten the free running period (*tau*) in male grey mouse lemurs [8]. 

Despite the importance of changes affecting the biological rhythms during aging, very few interventions are known to prevent such modifications. In humans, some studies using bright light therapy aimed to restore the circadian rhythms in elderly people. As an example, bright light therapy has been demonstrated as having beneficial effects on circadian rhythms in institutionalized elderly people [10]. Findings in animals allow us to propose nutritional interventions as another possible way to restore circadian rhythms during aging. Indeed, it was demonstrated that resveratrol (RSV), a natural compound found in grape and wine, regulates circadian clock genes in cultured Rat-1 fibroblast cells: a dose of 100 *μ*M RSV increased the amplitude of oscillation of clock genes Per1, Per2, and Bmal1 [11]. This last result suggests that dietary RSV might act as a regulator of circadian clocks. RSV is known and tested as a potential mimetic of chronic calorie restriction, an intervention that may prolong lifespan in several species of invertebrates and mammals [12]. RSV is also able to modify energy balance in several species from yeast to mice, by activating different proteins involved in energy regulation pathways, such as PGC1*α* [13] and SIRT1 [14], a nicotinamide adenosine dinucleotide-dependent deacetylase belonging to the sirtuins family. Interestingly, an important role of SIRT1 in biological clock processes has been identified [15, 16] so that the regulation of this protein by RSV could induce modifications of rhythm pattern of an organism's physiological parameters. In two recent studies on mouse lemurs, we demonstrated that RSV dietary supplementation was able to modify the endogenous period *tau* in young and aged animals [17] and was able to change the architecture of sleep-wake rhythms by lowering the amount of slow-wave sleep and increasing the proportion of activity in young animals [18].

Based on our previous findings, in the present study, we addressed the question whether RSV was able to restore the rhythms of daily locomotor activity and body temperature in aged mouse lemurs in comparison to young ones. 

## 2. Experimental Procedures

### 2.1. Animals and Animal Care

We used eight young adult (mean age: 26 ± 11 months old) and five aged (mean age: 76 ± 10 months old) female grey mouse lemurs (*Microcebus murinus*, Cheirogaleidae, Primates) born in a laboratory breeding colony in Brunoy, France (Agreement no. 962773) from a population originally caught 40 years ago on the southwest coast of Madagascar. Conditions were constant with respect to ambient temperature (25°C), relative humidity (55%), and *ad libitum* water availability. Behavioral and physiological seasonal changes of mouse lemurs are dependent on the photoperiod and are reproduced in captivity by an artificial photoperiodic regimen. In the breeding colony, animals were exposed to an artificial photoperiodic regimen consisting of alternating 6-month periods of Malagasy winter-like short-day lengths (L : D  10 : 14) and of Malagasy summer-like long-day lengths (L : D  14 : 10). A greater plasticity of the body temperature adjustments was observed in animals under short-day photoperiod with higher torpor depth and duration, respectively, than in animals under long-day photoperiod [19]. These results are consolidated by the observation that grey mouse lemur in the field enters torpor spontaneously during the dry season but not during the rainy period [20]. This is the reason why the animals used in this study were in short-day photoperiod. More particular, the animals were in the middle of their short-day photoperiod, just after their fattening phase, to avoid any perturbation of their physiological processes due to a lack of food resource. Young animals presented a mean body mass of 128 ± 11 g at the beginning of the experiment and of 156 ± 14 at the end of the experiment. Aged animals presented a mean body mass of 137 ± 10 g at the beginning of the experiment and of 152 ± 13 at the end of the experiment. The animals were weighed during the control week, the second week and the fourth week of experiment. To minimize social influences, the animals were housed individually in cages (0.4 × 0.4 × 0.6 m), provided with nesting materials, and separated from each other by wooden partitions. 

During the whole experiment, animals were fed with fresh fruits (banana and apple) and a mixture of cereals, milk, and eggs, providing them with a total of 120 kJ per day. The cereals are composed of 60% carbohydrates, 10% proteins, and 30% lipids. The cereals are primarily wheat flour (96%). After one control week to define basal levels of the different parameters analyzed in this study, the animals were fed, during the next 4 weeks (RSV1, RSV2, RSV3, and RSV4), with the same mixture as previous mentioned but 200 mg·kg^−1^ of RSV (Sequoia Research Products, United Kingdom) per day was added to the mixture. All the procedures were carried out in accordance with the European Communities Council Directive (86/609/EEC) and were done under personal licenses to experiment on mouse lemurs, delivered by the Ministry of Education and Science. Moreover, this study met the ethical standards of the journal [21].

### 2.2. Recording of Locomotor Activity and Body Temperature

Recording of locomotor activity (LA) and body temperature (Tb) was obtained by telemetry at a constant ambient temperature of 25°C. A small telemetric transmitter weighing 2.5 g (model TA10TA-F20, DataScience Co. Ltd, Minnesota, USA) was implanted into the visceral cavity under ketamine anesthesia (Imalgene, 100 mg/kg ip). After surgery, animals returned to their home cage and were allowed to recover for 15 days before start of experiment and continuous recordings of LA and Tb. Total recovery was checked by visual inspection of the complete healing of the surgical incision and by verification of a stable daily pattern of Tb variations. A receiver was positioned in the cage. Locomotor activity was continuously recorded by the receiver plate which detected vertical and horizontal movements (*X*-*Y* coordinate system, Dataquest Lab Pro v. 3.0, Data Science Co. Ltd, Minnesota, USA). LA data were summed in 5 min intervals and expressed in arbitrary unit (a.u.). The following parameters of LA have been defined: active phase LA (corresponding to the 8 most active hours of the dark period), LA onset (time between activity onset and the beginning of the dark period, expressed in min). LA onset was defined as the first 6 successive bins of 5 minutes in which activity was greater than the mean locomotor activity.

Tb was recorded every 10 min and allowed defining the following parameters: mean Tb during the dark phase (night Tb), mean Tb during the light phase (day Tb), and the minimal Tb reached during the hypothermia phase (Tb min). Entry into daily hypothermia was considered to start with the first value below 33°C, ending with the first value above 33°C (as defined by [19]). On this basis, the following hypothermia parameters were defined: hypothermia duration, reflecting the time during which Tb of an animal was under 33°C; hypothermia time drop (Hdrop), defined as the time from which mean Tb started to decrease (when 6 consecutive values were decreasing compared to the previous one); and time of minimal body temperature (Hmin), defined as the time at which Tb min was reached. For Hdrop and Hmin, the time of reference was the time of lights are on, with positive values before lights are on (phase advance) and negative values after lights on (phase delay). Tb was expressed in °C and hypothermia duration, Hdrop, and Hmin were expressed in min. LA and Tb data were averaged for each week of treatment (CTL, RSV1, RSV2, RSV3, and RSV4).

### 2.3. Statistics

All values are expressed as mean ± standard error to the mean. After checking for the normality of the distribution, ANOVA or repeated ANOVA for related samples was used to assert significant variations in locomotor activity, body temperature, and daily hypothermia parameters. Data from hypothermia duration were log-transformed in order to obtain data with a normalized distribution. Comparisons were considered to differ significantly when *P* < 0.05. All statistical computations were performed using SYSTAT for Windows (V9, SPSS Inc., USA).

## 3. Results

### 3.1. Locomotor Activity Parameters

**Figure 1:**
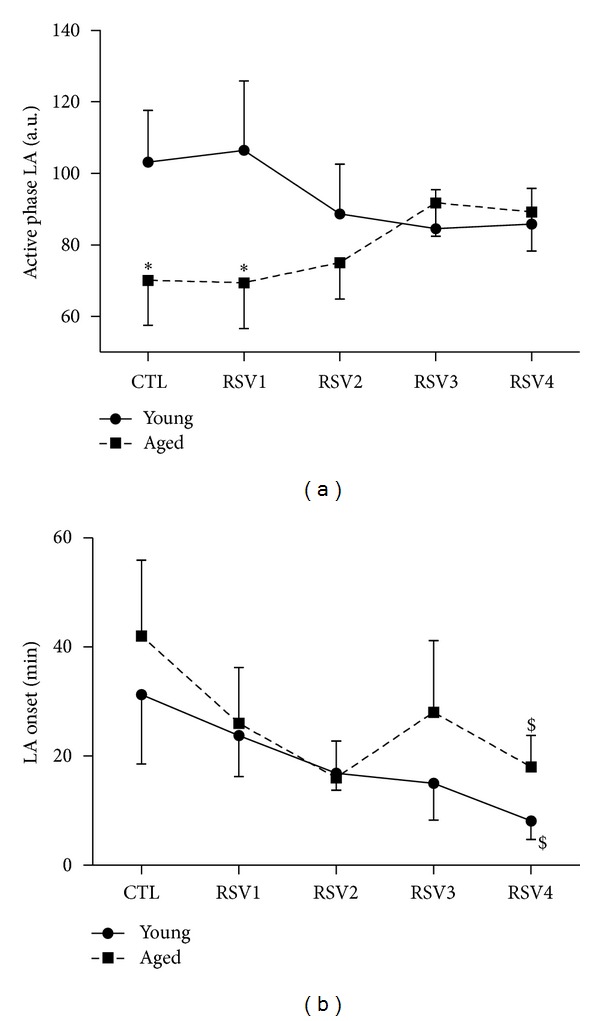
Locomotor activity (LA) parameters in young (solid lines) and aged (dotted lines) female mouse lemurs. (a) LA during the active phase is expressed in arbitrary units (a.u.). (b) LA onset is expressed in min. During the first week, animals received the control feeding (CTL), followed by 4 weeks of resveratrol (RSV) supplementation. Values are given as mean ± standard error of the mean. *represents a significant difference between young and aged animals (*P* < 0.05). ^$^represents a significant effect of RSV supplementation compared to CTL condition (*P* < 0.05).

Active phase LA was significantly higher in young animals compared to aged animals during the CTL and the first week of RSV supplementation (*P* = 0.007, Figure 1(a)). From RSV2 to RSV4 weeks, young and aged animals exhibited similar levels of active phase LA. LA onset was not significantly different between young and aged animals at the beginning of the study. Indeed, during CTL condition, a phase advance of 31 ± 13 min was observed for young animals and 42 ± 14 min for aged animals (Figure 1(b)). During the 4 weeks of RSV supplementation, young and aged animals then exhibited comparable reduction of the phase advance of LA onset to reach 8 ± 3 min and 18 ± 6 min respectively, at the end of RSV supplementation (*P* = 0.03, Figure 1(b)).

### 3.2. Body Temperature

**Figure 2:**
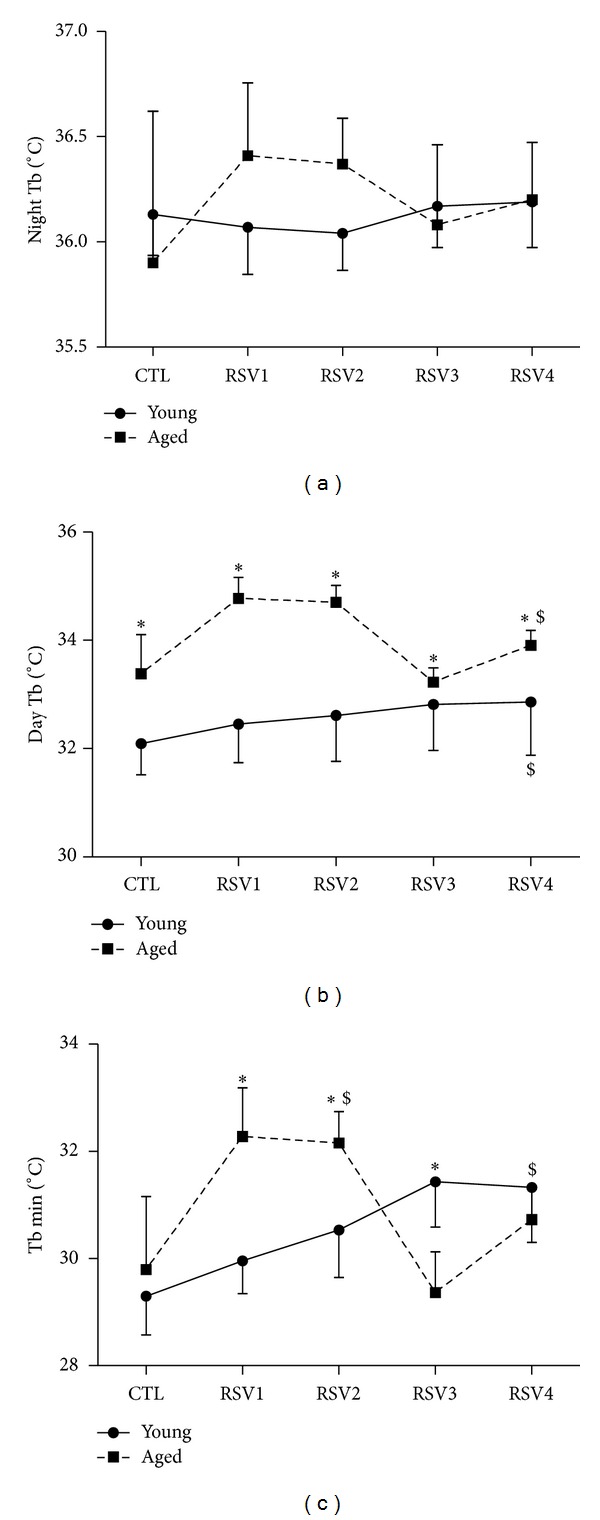
Body temperature (Tb) parameters in young (solid lines) and aged (dotted lines) female mouse lemurs. (a) Mean Tb during the dark phase. (b) Mean Tb during the light phase. (c) Lower Tb (Tb min) reached during hypothermia phase. During the first week, animals received the control feeding (CTL), followed by 4 weeks of resveratrol (RSV) supplementation. Tb was expressed in °C. Values are given as mean ± standard error of the mean. *represents a significant difference between young and aged animals (*P* < 0.05). ^$^represents a significant effect of RSV supplementation compared to CTL condition (*P* < 0.05).

Night Tb during CTL condition was similar in young and aged animals (36.1 ± 0.2°C and 35.9 ± 0.3°C, resp.) (Figure 2(a)). No significant change in night Tb occurred during the 4 weeks of treatment in animals of both age groups. Conversely, significant differences and variations in day Tb have been observed (Figure 2(b)). Under CTL condition, mean day Tb was 32.1 ± 0.6°C in young animals and 33.4 ± 0.7°C in aged animals, a difference that was statistically significant (*P* = 0.004). During RSV supplementation, day Tb significantly increased in both age groups, reaching 32.8 ± 1.0°C in young animals (*P* = 0.04) and 33.9 ± 0.3°C in aged animals (*P* = 0.03). Despite a transient and significant decrease of day Tb in aged animals during the third week of RSV supplementation, day Tb remained significantly higher in aged animals compared to young ones during the whole experiment (*P* = 0.002). Tb min, which was reached during the daily hypothermia, was comparable between young and aged animals at the start of the experiment (CTL condition) and then followed a similar pattern as day Tb (Figure 2(c)). Young animals exhibited an increase in Tb min from CTL condition (29.3 ± 0.7°C) to RSV4 (31.3 ± 1.0°C) (*P* = 0.02). Aged animals first exhibited an increase of Tb min from CTL condition (29.8 ± 1.4°C) to RSV2 condition (*P* = 0.04); then at RSV3, similarly to day Tb, they exhibited a transient decrease of Tb min. At RSV4, Tb min in aged animals reached similar values (30.7 ± 0.6°C) compared to those in young animals (31.3 ± 1.0°C).

### 3.3. Daily Hypothermia Parameters

**Figure 3:**
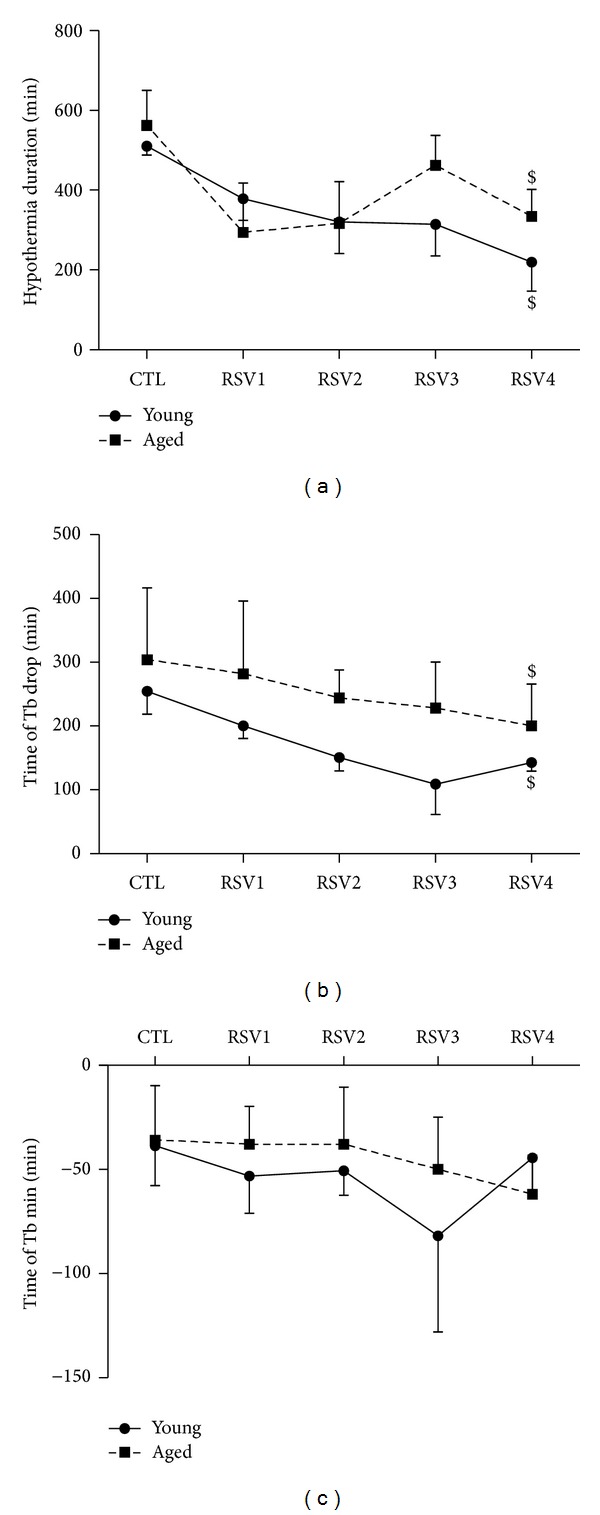
Daily hypothermia parameters in young (solid lines) and aged (dotted lines) female mouse lemurs. (a) Hypothermia duration (expressed in min). (b) Time from which the mean body temperature (Tb min) of the day decreased (Hdrop). (c) Time at which Tb min was reached (Hmin). During the first week animals received the control feeding (CTL), followed by 4 weeks of resveratrol (RSV) supplementation. Values are given as mean ± standard error of the mean. *represents a significant difference between young and aged animals (*P* < 0.05). ^$^represents a significant effect of RSV supplementation compared to CTL condition (*P* < 0.05).

Hypothermia duration was similar between young and aged animals and changed similarly during the experiment. Hypothermia duration during CTL condition was 510 ± 23 min in young animals and 562 ± 88 min in aged animals. It significantly decreased to 219 ± 73 min for young animals (*P* = 0.02) and 334 ± 67 min for aged animals during RSV4 condition (*P* = 0.04, Figure 3(a)). Time of Tb drop (Hdrop) was not significantly different between young and aged animals (*P* = 0.6) and significantly decreased similarly in both age groups (*P* = 0.04 in both young and aged animals) (Figure 3(b)). Time at which Tb min was reached (Hmin) was not different between young and aged animals (*P* = 0.9) and did not change during the treatment (*P* = 0.8 and *P* = 0.4, resp., for young and aged animals) (Figure 3(c)).

## 4. Discussion

In the present study, we investigated the impact of RSV dietary supplementation on the rhythms of daily locomotor activity and body temperature in young and aged mouse lemurs. 

RSV supplementation induced a reduction of LA onset suggesting a better synchronization with lights off. Young animals also exhibited a gradual increase in day Tb and Tb min under RSV supplementation. This increase in body temperature also led to a significant decrease in hypothermia duration after 4 weeks of RSV supplementation. In the same way, Hdrop was significantly delayed, leading to better synchronization with lights on and shortening of the torpor duration. These results are consistent with our previous observations in male grey mouse lemur in which a 4-week RSV supplementation inhibited the depth of daily torpor and significantly increased day Tb and Tb min [22]. Changes in LA onset and Hdrop confirm the specific impact of dietary RSV on circadian clock parameters previously observed by our group. Indeed, we observed that grey mouse lemurs in constant dark conditions (free-running experiments) exhibited a shortening of their endogenous period under RSV supplementation compared to controls [17].

In aged animals, active phase LA was lower in CTL condition compared to young animals. After 4 weeks of RSV supplementation, active phase LA reached similar values in aged and in young mouse lemurs. It is noteworthy that LA activity is affected differently by RSV supplementation in young and aged animals. It is widely described that aged animals [7, 8] and humans [10] exhibit a spontaneous impairment in their circadian rhythms that leads to increased resting phase LA and decreased active phase LA, showing the frailty of the aged individuals circadian clock. Even if no mechanism can be proposed at this time to explain the differential effect of RSV on young and aged animals, it is highly probable that this is due to the frailty of aged individual's circadian clock. Interestingly, under RSV conditions, we observed the same reduction of LA onset in aged animals as in young ones, suggesting a better synchronization with lights off in both age groups. Changes in locomotor activity parameters under RSV supplementation in aged animals suggest a restoration of some circadian rhythm parameters similar to those observed in young animals. 

Day Tb remained significantly higher in aged animals compared to that in young ones during the course of the experiment ones and, similar to young animal, aged animals exhibited a gradual increase in day Tb with RSV supplementation. Tb min followed a similar pattern as day Tb with a final Tb min increase at the end of the 4 weeks of RSV supplementation. Similarly to young, hypothermia duration significantly decreased for aged animals during RSV4 condition and Hdrop was also significantly delayed. The shortening of torpor duration and higher day Tb suggest a specific impact of RSV on energy metabolism. We previously observed a similar difference in the impact of RSV on young or aged animals, with the endogenous period *tau* of aged animals being significantly more reduced by RSV supplementation than the one of young animals [17]. These observations suggest that aged animals, known to exhibit impaired circadian rhythms [7, 8], may respond better to RSV positive effects, leading to an almost partial restoration of the rhythms, compared to young ones. 

The results of the present study suggest that RSV might act as a potent regulator of circadian rhythms, more particularly during aging. Strong relations between metabolism and the regulation of the circadian clock have been recently evidenced [23]. Some studies suggest that relations between metabolism and circadian rhythms could be driven by changes in the expression of clock genes [24]. Caloric restriction, a regime known to prolong lifespan in various species [25], also affects circadian rhythms [26, 27] probably via activation of SIRT1. Since it has been demonstrated that RSV was able to activate the transcription of SIRT1 [14], which directly binds to the CLOCK/BMAL1 complex to regulate expression of clock genes [15, 16, 28, 29], the beneficial effects exerted by RSV could be mediated through resetting of the circadian clock, thus leading to better synchrony in metabolism and physiology [30]. However, it is noteworthy that the RSV-induced SIRT1 activation is now under debate [31]. Further studies focusing on the impact of RSV on clock and metabolism-related genes are now needed to address this hypothesis.

These results confirm the specific impact of RSV on rhythms parameters, with a more marked effect in aged animals (active phase LA and day Tb). The results of this study suggest that RSV might act as a potent regulator of both circadian clock and metabolism. RSV supplementation might thus represent a new and promising nonpharmacological treatment of circadian perturbations associated with normal or pathological aging such as Alzheimer's disease [32].
